# Lycorine suppresses RANKL-induced osteoclastogenesis *in vitro* and prevents ovariectomy-induced osteoporosis and titanium particle-induced osteolysis *in vivo*

**DOI:** 10.1038/srep12853

**Published:** 2015-08-04

**Authors:** Shuai Chen, Gu Jin, Kang-Mao Huang, Jian-Jun Ma, Qiang Wang, Yan Ma, Xiao-Zhen Tang, Zhi-Jie Zhou, Zhi-Jun Hu, Ji-Ying Wang, An Qin, Shun-Wu Fan

**Affiliations:** 1 Department of Orthopaedics, Sir Run Run Shaw Hospital, School of Medicine, Zhejiang University, Hangzhou 310016, China; 2 Key Laboratory of Biotherapy of Zhejiang Province, Hangzhou 310016, China; 3 Department of Orthopaedics, Shanghai Key Laboratory of Orthopaedic Implant, Shanghai Ninth People’s Hospital, Shanghai Jiaotong University School of Medicine, Shanghai 200011, China; 4 Department of Bone and Soft Tissue Surgery, Zhejiang Cancer Hospital, Hangzhou 310022, China

## Abstract

Osteoclasts play an important role in diseases involving bone loss. In this study, we assessed the effect of a plant-derived natural alkaloid (lycorine, or LY) on osteoclastogenesis *in vitro* and *in vivo*. Our *in vitro* study showed that receptor activator of nuclear factor-κB ligand (RANKL)-induced osteoclastogenesis could be inhibited by LY; this effect was due to inhibition of mitogen-activated protein kinase (MAPK) signalling via MAP kinase kinases (MKKs). The MAPK agonist anisomycin could partially rescue the inhibitory effect of LY. Furthermore, LY also played a protective role in both a murine ovariectomy (OVX)-induced osteoporosis model and a titanium particle-induced osteolysis model. These results confirmed that LY was effective in preventing osteoclast-related diseases *in vivo*. In conclusion, our results show that LY is effective in suppressing osteoclastogenesis and therefore could be used to treat OVX-induced osteoporosis and wear particle-induced osteolysis.

Bone homeostasis is dynamically balanced by osteoclastic bone resorption and osteoblastic bone formation. Osteoclasts are the only cells capable of bone resorption, and excessive osteoclast activity can lead to osteolytic diseases such as osteoporosis and periprosthetic osteolysis. Generally, osteoclasts are multinucleated cells that originate from the haematopoietic progenitors of the monocyte/macrophage lineage. The differentiation of progenitors into osteoclasts requires critical cytokines, including receptor activator of nuclear factor-κB ligand (RANKL) and macrophage colony-stimulating factor (M-CSF)^1^,^2^. RANKL is the key cytokine for osteoclast differentiation, while M-CSF is necessary for the survival and proliferation of osteoclast precursors^3^. Binding of RANKL to its receptor RANK leads to the trimerization of RANK and the recruitment of adaptor molecules, such as tumour necrosis factor receptor-associated factor 6 (TRAF6)^4^, followed by the activation of signalling pathways, including the mitogen-activated protein kinase (MAPK) and nuclear factor kappaB (NF-κB) pathways^5^,^6^. Downstream regulators such as c-Fos^7^ and nuclear factor of activated T cells c1 (NFATc1)^8^ are subsequently upregulated and activated, resulting in the formation of osteoclasts. Therefore, key cytokines, along with RANKL-induced signalling pathways, are potential molecular targets for the treatment of osteoclastic osteolysis diseases. Indeed, use of the RANKL neutralizing antibody denosumab has proven to be a potential treatment for osteoporosis and tumour-induced osteolysis^9^.

In addition to antibodies such as denosumab, natural compounds exhibit good anti-resorption properties and have great potential for inhibiting RANKL-induced osteoclastogenesis and osteolytic diseases^10^,^11^,^12^,^13^. Our group has had a long interest in screening natural compounds for osteolysis diseases^14^,^15^,^16^. During this screening process, we identified lycorine (LY) as a novel anti-resorption compound. LY is a natural alkaloid that exists in the flowers and bulbs of Amaryllidaceae species, such as daffodils (*Narcissus*), snowdrops (*Galanthus*) and spider lilies (*Lycoris*)^17^. LY has been shown to possess powerful anti-inflammatory^18^, anti-viral^19^ and anti-malarial^20^ properties in previous studies. LY has also demonstrated a potential anti-tumour effect, with low toxicity, due to its suppression of tumour cell growth and induction of apoptosis in ovarian cancer^21^, myeloid leukaemia^22^ and melanoma^23^. Additionally, Kang *et al.*^24^ showed that LY inhibited lipopolysaccharide (LPS)-induced P38 pathway activation in the RAW264.7 macrophage cell line.

Given the importance of MAPK signalling pathways in osteoclast formation, the possible effect of LY on P38 suppression in a macrophage cell line, and the wide clinical application of LY, we hypothesized that LY may represent a novel treatment for osteoclast-related diseases. Therefore, in this study, we aimed to (i) investigate the effect of LY on osteoclastogenesis; (ii) elucidate the underlying mechanisms that mediate the effects of LY on osteoclast formation and function; and (iii) test the therapeutic effect of LY in a murine ovariectomy (OVX)-induced bone loss model, as well as a titanium (Ti) particle-induced osteolysis model.

## Results

### LY suppressed RANKL-induced osteoclast differentiation without cytotoxicity *in vitro*

**Figure 1:**
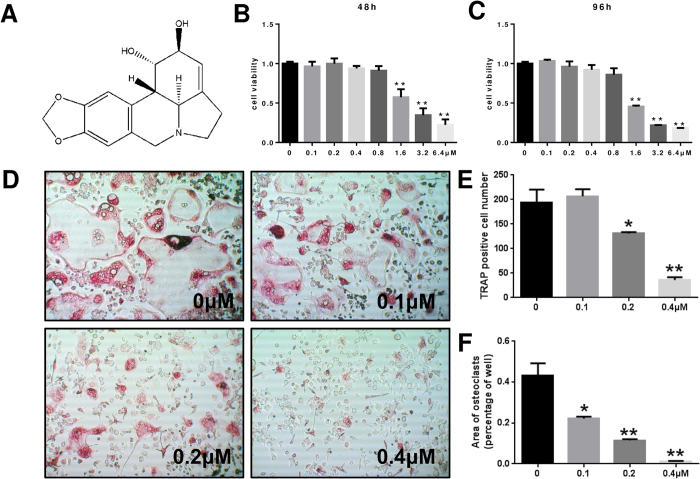
LY inhibited RANKL-induced osteoclastogenesis without cytotoxic effects *in vitro*. (**A**) The structure of LY. (**B**,**C**) Viability of LY-treated BMMs tested by CCK8 assays at 48 and 96 h. (**P < 0.01). (**D**) BMMs were treated with various concentrations of LY, M-CSF (30 ng/mL) and RANKL (50 ng/mL) for 5 days. Then, the cells were fixed with 4% paraformaldehyde and stained for TRAP. (**E**,**F**) The number and areas of TRAP-positive multinuclear cells were determined as described in the Methods section (*P < 0.05; **P < 0.01).

We first tested the effect of LY on osteoclast precursor cells. Specifically, we tested primary bone marrow macrophage (BMM) cell viability for 48 h and 96 h to determine which doses produced a toxic effect. There were no cytotoxic effects of LY at doses below 0.8 μM. The IC50 values for LY were 1.320 μM (48 h) and 1.577 μM (96 h) (Fig. 1B,C). To determine the effect of LY on RANKL-induced osteoclast differentiation, we treated BMMs with RANKL and M-CSF in the presence of 0, 0.1, 0.2 or 0.4 μM of LY. As shown in Fig. 1D, LY significantly inhibited the formation of tartrate-resistant acid phosphatase (TRAP)-positive multinucleated osteoclasts in a dose-dependent manner. The TRAP-positive cell number decreased from 194.4/well (LY 0 μM) to 36.5/well (LY 0.4 μM), and the total osteoclast area dropped from 43.3% (LY 0 μM) to 1.1% (LY 0.4 μM) (Fig. 1E,F). Collectively, these data showed that LY could effectively inhibit osteoclastogenesis without a cytotoxic effect.

### LY inhibited osteoclastic bone resorption *in vitro*

**Figure 2:**
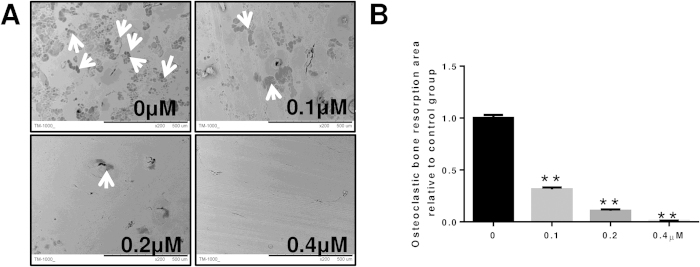
LY inhibited osteoclastic bone resorption *in vitro*. BMMs were seeded onto bone slices and treated the same as described in Fig. 1C for 7 days. (**A**) Scanning electron microscope (SEM) images of bone resorption pits are shown. (**B**) Resorption pit areas were measured using Image J. (**P < 0.01).

To further investigate the role of LY in osteoclastic bone resorption, BMMs plated on bovine bone slices were treated with 0, 0.1, 0.2 or 0.4 μM of LY. LY treatment substantially reduced the area of bone resorption, as shown in Fig. 2A. The bone resorption area of slices treated with 0.1 μM LY was 30% less than the control group. However, bone resorption was almost completely abolished after treatment with 0.4 μM LY (Fig. 2B). Together, these data showed that LY inhibited osteoclast bone resorption *in vitro*.

### LY inhibited RANKL-induced osteoclast-specific gene expression *in vitro*

**Figure 3:**
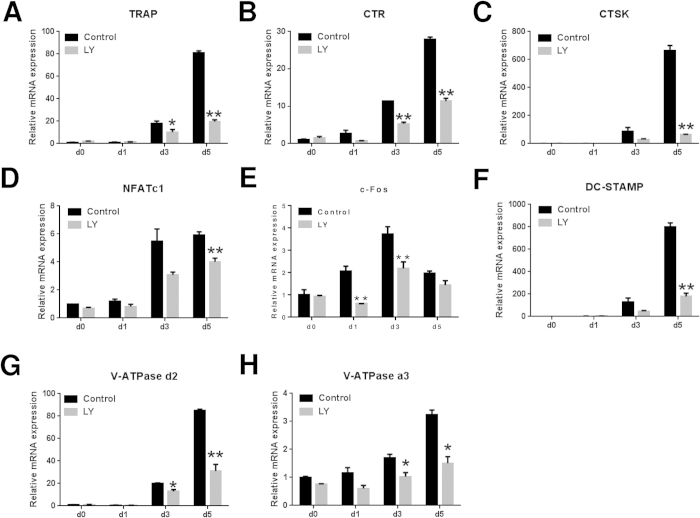
LY inhibited RANKL-induced osteoclast-specific gene expression *in vitro*. (**A**–**H**) Expression of the osteoclast-specific genes TRAP, CTR, CTSK, NFATc1, c-Fos, DC-STAMP, V-ATPase d2, and V-ATPase a3 in BMMs treated with LY (0.4 μM), M-CSF (30 ng/mL) and RANKL (50 ng/mL) for 0, 1, 3 or 5 days. Gene expression was analysed by real-time PCR. RNA expression levels were normalized relative to the expression of GAPDH (*P < 0.05; **P < 0.01).

To further assess the inhibitory effects of LY on osteoclastogenesis, we examined the RANKL-induced mRNA expression profiles of osteoclast-specific genes, including TRAP, calcitonin receptor (CTR), cathepsin K (CTSK), c-Fos, NFATc1, V-ATPase-d2, V-ATPase-a3 and dendritic cell-specific transmembrane protein (DC-STAMP). As demonstrated in Fig. 3A–H, these genes were significantly upregulated during the formation of osteoclasts for 1, 3 or 5 days. In contrast, the expression of these genes was significantly suppressed after LY treatment.

### LY suppressed the MAPK signalling pathway during osteoclastogenesis *in vitro*

**Figure 4:**
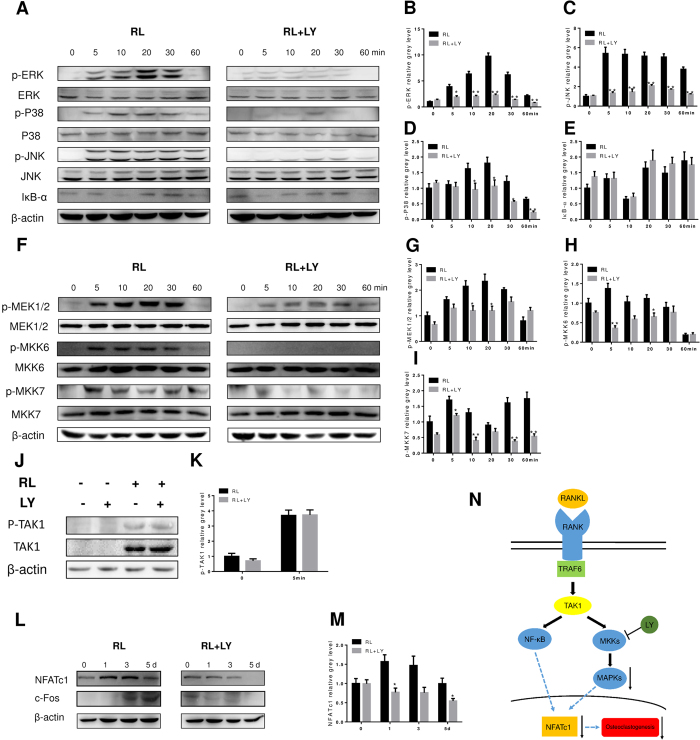
LY inhibited osteoclastogenesis by impairing RANKL-induced MAPK pathways without affecting the NF-κB pathway *in vitro*. (**A**,**F**) BMMs were treated with or without 0.4 μM LY for 4 h and then treated with 100 ng/mL RANKL (RL) for the indicated periods. Cell lysates were analysed using western blotting. The expression of phosphorylated ERK, P38, JNK, total ERK, P38, and JNK, IκB-α (A), phosphorylated MEK1/2, MKK6, MKK7, and total MEK1/2, MKK6, MKK7, and β-actin was evaluated; (**B**–**E**) The grey levels of phosphorylated ERK, P38, and JNK were quantified and normalized to total ERK, P38, and JNK using Image J. The grey level of IκB-α was normalized to β-actin (*P < 0.05; **P < 0.01). (**G**–**I**) The grey levels of phosphorylated MEK1/2, MKK6, and MKK7 were quantified and normalized to total MEK1/2, MKK6, and MKK7 using Image J (*P < 0.05; **P < 0.01). (**J**) BMMs were treated with or without 0.4 μM LY for 4 h and then treated with or without 100 ng/mL RANKL for 5 min. Cell lysates were analysed using western blotting. The expression of phosphorylated TAK1, total TAK1, and β-actin was evaluated. (**K**) The grey levels of phosphorylated TAK1 were quantified and normalized to β-actin using Image J. (**L**) BMMs were treated with RANKL, with or without 0.4 μM LY, for the indicated periods. Cell lysates were analysed using western blotting. The expression of NFATc1 and β-actin was evaluated. (**M**) The grey levels of NFATc1 were quantified and normalized to β-actin using Image J (*P < 0.05). (**N**) Schematic diagram of the mechanism by which LY inhibits osteoclast differentiation and function.

To elucidate the mechanisms by which LY affects osteoclastogenesis, we investigated key signalling pathways, including the MAPK signalling pathway and the NF-κB pathway. BMMs were treated with RANKL, with or without LY, for 0, 5, 10, 20, 30 and 60 min. As shown in Fig. 4A, LY significantly attenuated extracellular signal-regulated kinase (ERK), P38, and c-Jun N-terminal kinase (JNK) phosphorylation. However, the levels of nuclear factor of kappa light polypeptide gene enhancer in B-cells inhibitor alpha (IκB-α) were unchanged after LY treatment (Fig. 4A). These observations were confirmed by quantitative analysis (Fig. 4B–E). These data suggested that LY inhibited the MAPK signalling pathway without affecting NF-κB activation. To further explore whether LY directly blocked P38, JNK, or ERK signalling, we examined the activation of upstream regulators, including MAP kinase kinase 1/2 (MEK1/2), MAP kinase kinase 6 (MKK6), and MAP kinase kinase 7 (MKK7) in BMMs treated in the same manner as described above. The results showed that the phosphorylation of MKKs was also attenuated after LY treatment (Fig. 4F–I). To confirm the exact targets of LY, we assessed the phosphorylation of transforming growth factor β-activated kinase 1 (TAK1), which is the up-stream kinase of MKKs. Interestingly, the phosphorylation of TAK1 was unchanged when BMMs were treated with RANKL and LY, in comparison to cells treated with RANKL alone (Fig. 4J,K), indicating that MKKs are possible targets of LY. Because NFATc1 is important for initiating osteoclast differentiation, we also tested the expression of NFATc1 in BMMs treated with RANKL and LY for 0, 1, 3 or 5 days. As expected, western blot analysis showed that RANKL-induced expression of NFATc1 was significantly reduced following LY treatment (Fig. 4L). This observation was also confirmed by quantitative analysis (Fig. 4M). Taken together, these data suggested that LY inhibited the MAPK pathway by targeting MKKs, without affecting the NF-κB signalling pathway, as described in Fig. 4N.

### The P38 and JNK agonist anisomycin partially rescued the inhibition of osteoclastogenesis by LY *in vitro*

**Figure 5:**
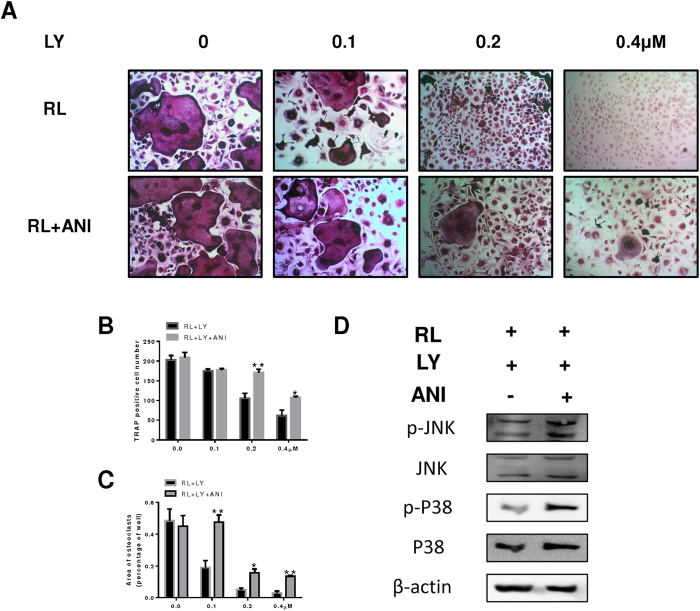
Anisomycin (ANI) attenuated the inhibitory effect of LY on osteoclastogenesis *in vitro*. (**A**) BMMs were stimulated with 30 ng/mL M-CSF, 50 ng/mL RANKL, and LY (0, 0.1, 0.2 or 0.4 μM), with or without 2.5 pg/mL ANI, for 5 days. All cells were fixed and stained for TRAP as described in the Methods section. (**B**,**C**) The number and areas of TRAP-positive cells were determined using Image J (*P < 0.05; **P < 0.01). (**D**) BMMs were treated with 0.4 μM LY for 4 h and then treated with 100 ng/mL RL, with or without 2.5 pg/mL ANI, for 5 min. Cell lysates were analysed using western blotting. The expression of phosphorylated P38 and JNK, total P38 and JNK, and β-actin was evaluated.

As stated above, osteoclast formation was greatly inhibited by LY due to the reduced activation of MAPK signalling pathways. To confirm these results, we treated BMMs with a strong agonist of the JNK and P38 pathways (anisomycin) following treatment with LY. As shown, osteoclast formation was inhibited in cells treated with LY, whereas in cells also treated with anisomycin, the impaired osteoclastogenesis was partially rescued and mature osteoclasts were observed (Fig. 5A). Analysis of osteoclast number and area confirmed this result (Fig. 5B,C). The inhibition of P38 and JNK phosphorylation following LY treatment was also reversed in BMMs treated with LY and anisomycin (Fig. 5D).

### Administration of LY prevented OVX-induced bone loss *in vivo*

**Figure 6:**
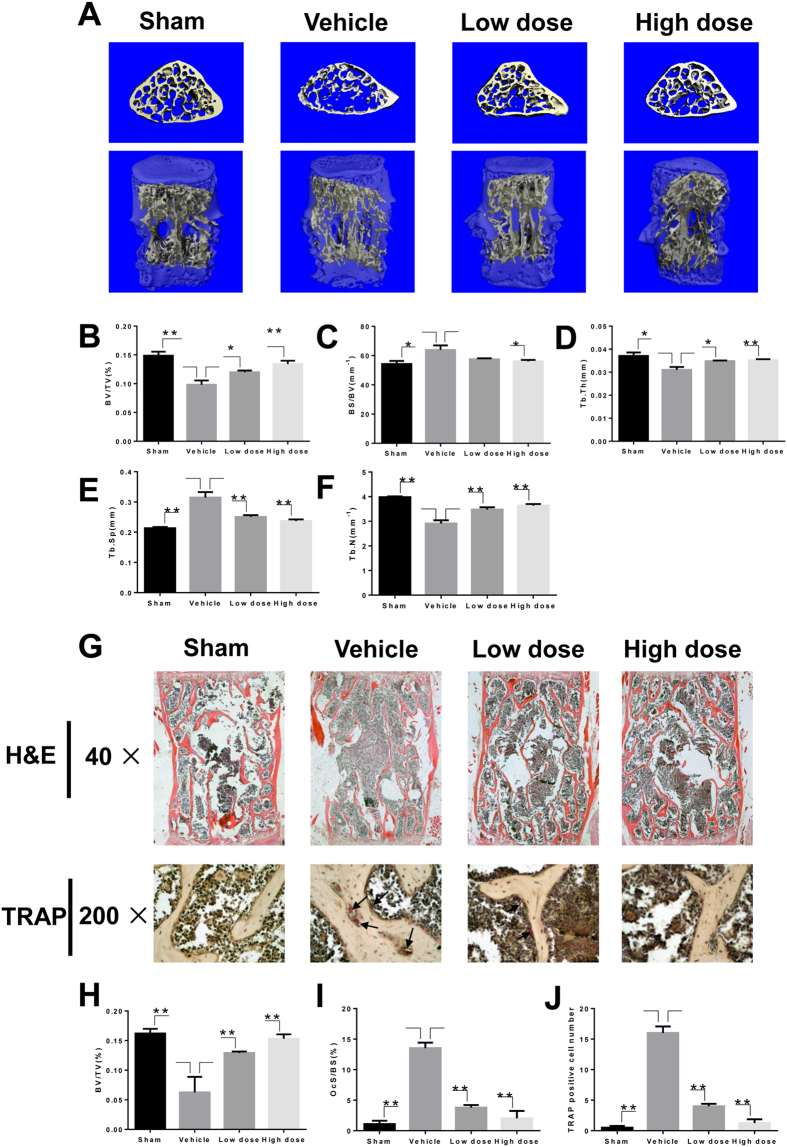
LY effectively prevented OVX-induced bone loss *in vivo*. (**A**) The lumbar vertebral bodies (L5) of all mice were scanned with a high-resolution micro-CT. (**B**–**F**) Calculation of the microstructural indices was performed for the micro-CT data as described in the Methods section. Microstructural indices include bone volume/tissue volume (BV/TV), bone surface/bone volume (BS/BV), trabecular separation (Tb.Sp.), trabecular thickness (Tb.Th.), and trabecular number (Tb.N.) (*P < 0.05; **P < 0.01). (**G**) The vertebral bodies of L3-L5 were fixed in 4% paraformaldehyde, decalcified, embedded, and sectioned as described in the Methods section. Sections of vertebral bodies were stained with H&E (40×) and TRAP (200×). (**H**) BV/TV was measured in sections stained with H&E (**P < 0.01). (**I**,**J**) The percentage of osteoclast surface per bone surface (OcS/BS%) and the number of osteoclasts per field of tissue in sections stained by TRAP in 200× magnification were analysed (**P < 0.01).

**Figure 7:**
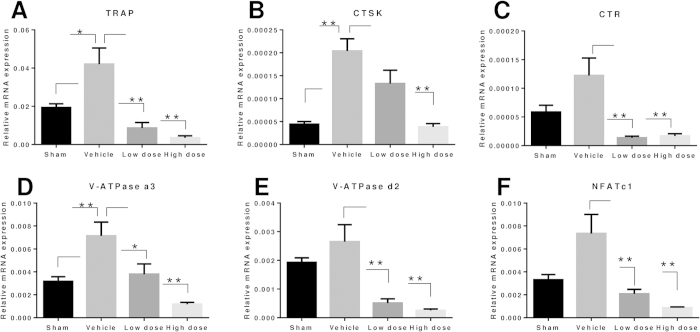
LY prevention of OVX-induced bone loss assessed by RT-PCR. (**A**–**F**) Total RNA was extracted from the left femur and tibia of each mouse and reverse transcribed into cDNA, which was used in RT-PCR as described in the Methods section. The expression levels of TRAP, CTSK, CTR, V-ATPase a3, V-ATPase d2, and NFATc1 were examined (*P < 0.05; **P < 0.01).

To explore the preventive effects of LY on osteoporosis, we further examined LY treatment in an OVX-induced murine osteoporosis model. The decreased uterine weight and increased body weight of groups treated with OVX confirmed that the model was successfully established (Fig. S1A and B). Extensive bone loss in vertebral bodies of the lumbar spine was observed by micro-computed tomography (CT) in the vehicle-treated group. Bone volume/tissue volume (BV/TV), bone surface/bone volume (BS/BV), trabecular thickness (Tb.Th), trabecular number (Tb.N), and trabecular separation (Tb.Sp) were measured from the three-dimensional (3D) reconstructed images. Compared with the sham group, OVX significantly decreased the values of BV/TV, Tb.N, and Tb.Th and increased the values of Tb.Sp and BS/BV in lumbar vertebral bodies (Fig. 6B–F). In contrast, increased bone mass was observed in the groups treated with LY compared to vehicle alone (Fig. 6A). The evaluation of microstructural indices confirmed this observation (Fig. 6B–F). Further histological analysis confirmed the protective effect of LY on OVX-induced bone loss. Haematoxylin and eosin (H&E) staining showed severely reduced trabecular numbers and thicknesses in the vehicle-treated group and reservation of bone mass in LY-treated groups (Fig. 6G,H). Furthermore, TRAP staining showed that multinucleated osteoclasts were significantly increased in the vehicle-treated group but were reduced in the LY-treated groups (Fig. 6G,I,J). Similar results were demonstrated in tibiae by micro-CT and histological analyses (Fig. S1 C-I). Transcriptional levels of osteoclastogenesis-related genes were also upregulated in the vehicle-treated group and downregulated in a dose-dependent manner in the LY-treated groups (Fig. 7A–F).

### Administration of LY prevented Ti particle-induced osteolysis *in vivo*

**Figure 8:**
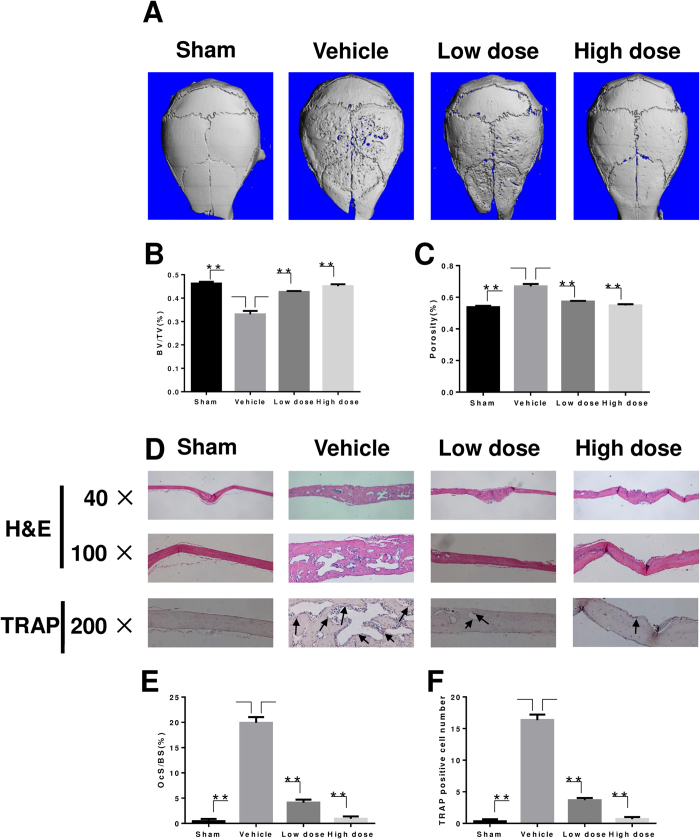
LY prevention of Ti particle-induced mouse calvarial osteolysis assessed by micro-CT. (**A**) Representative micro-CT 3D reconstructed images were obtained for each group. (**B**,**C**) The BV/TV and porosity of each sample was measured (**P < 0.01). (**D**) Assessment of LY prevention of Ti particle-induced mouse calvarial osteolysis was conducted with H&E staining and TRAP staining. (**E**,**F**) The OcS/BS and number of osteoclasts per field of tissue in 200× magnification were analysed (**P < 0.01).

To determine the preventive effect of LY on wear particle-induced osteolysis, we developed a Ti particle-induced calvarial osteolysis model. Mice were injected with LY for 2 weeks. Based on micro-CT scans and 3D reconstruction, we found that severe bone resorption occurred in the vehicle-treated group compared with the sham group (Fig. 8A). However, treatment with LY suppressed Ti particle-induced osteolysis in a dose-dependent manner. Furthermore, bone resorption in the high-dose group was significantly lower compared to that observed in the low-dose group (Fig. 8A). Quantification of bone parameters confirmed that the low-dose group and the high-dose group showed significantly increased BV/TV values and reduced porosity (Fig. 8B,C). Histological assessment further confirmed that LY treatment protected the mice against Ti particle-induced bone loss. H&E staining revealed that osteolysis had clearly occurred in sections obtained from the vehicle group, whereas the LY-treated groups exhibited significantly reduced osteolysis (Fig. 8D). Furthermore, the results of TRAP staining were in accordance with the results of micro-CT and H&E staining and revealed that the number of osteoclasts in the erosion site was increased in the vehicle-treated group (Fig. 8D). However, in both LY-treated groups, the number of osteoclasts and the osteoclast surface/bone surface area (OcS/BS) were decreased (Fig. 8E,F), indicating that LY treatment inhibited osteoclast formation and prevented osteoclastic bone loss during Ti particle-induced osteolysis *in vivo*.

## Discussion

Osteoclasts are the primary cells involved in bone resorption and cannot be replaced by other cell types^25^,^26^. Elevated numbers and enhanced activities of osteoclasts may lead to diseases related to excessive bone resorption, including wear particle-induced osteolysis, osteoporosis, rheumatoid arthritis, multiple myeloma, and metastatic cancers^1^,^25^,^27^. Thus, it is reasonable to consider osteoclasts as an effective target for the prevention or treatment of osteolytic diseases. Treatments targeting osteoclasts include oestrogen-replacement therapy, bisphosphonates (BPs), and denosumab^28^. These therapies have been shown to preserve or improve bone mass and reduce the risk of fracture^29^,^30^,^31^. However, oestrogen-replacement therapy can increase the risk for breast cancer, stroke, and heart attack^32^,^33^, while prolonged use of BPs has been reported to be correlated with osteonecrosis of the jaw (ONJ) and atypical fractures^34^. Although the humanized anti-RANKL neutralizing monoclonal antibody denosumab has been shown be an efficient, safe, and cost-effective treatment for osteoclast-related diseases^28^,^35^, its long-term efficacy remains to be confirmed.

In our study, we have demonstrated for the first time *in vitro* that plant-derived LY could prevent osteoclastogenesis by blocking the MAPK signalling pathway. Our results are also the first to show that LY could preserve bone mass *in vivo* in an OVX-induced bone loss murine model and wear particle-induced osteolysis model. Thus, our study identified LY as a potential new drug for the treatment of osteoporosis and wear particle-induced periprosthetic osteolysis, and our results contribute to knowledge on the mechanism of action of LY for further applications and research.

RANKL-induced osteoclast differentiation is mediated by downstream signalling pathways, including the P38, ERK, JNK, and NF-κB pathways. The P38, ERK and JNK pathways comprise the MAPK signalling pathway. The serine and threonine residues of P38, ERK, and JNK are phosphorylated by upstream kinases, and the signals are transmitted to the nucleus in order to regulate downstream molecules^36^. ERK is reported to induce the expression of downstream molecules driving osteoclastogenesis^7^, and blockade of the ERK pathway has been shown to decrease osteoclast formation^37^. P38 is involved in the early stages of osteoclast differentiation by regulating the microphthalmia-associated transcription factor^38^. Phosphorylation of JNK can modulate the transcriptional activity of activator protein-1 (AP-1), which is an important transcription factor in osteoclastogenesis^39^. Our results showed that LY could inhibit the phosphorylation of all three MAPKs in osteoclast precursor cells. Because LY did not influence the activation of TAK1, but significantly inhibited the phosphorylation of MKKs, we propose that MKKs are the targets of LY. Influence of LY on the activation of NF-κB was not observed, as LY did not affect the degradation of IκB-α. To confirm that LY acts through inhibition of the MAPK signalling pathway, we used an activator of JNK and P38 (anisomycin) to treat BMM cells after incubation with LY. As expected, suppressed phosphorylation of P38 and JNK could be reversed, and impaired osteoclastogenesis could be partially rescued. Kang *et al.* demonstrated that LY inhibited the LPS-induced upregulation of iNOS and COX-2 through suppression of the P38 and STAT pathways without influencing the ERK and JNK pathways in the RAW264.7 macrophage cell line^24^. Because the downstream cascades are activated in both a MyD88-dependent and -independent manner by LPS/Toll-like receptor 4 (TLR4)^40^, and the MAPK and NF-κB pathways are activated by RANKL through key factors such as TRAF6 and TAK1^1^, the mechanistic difference between the results reported by Kang *et al.* and our study is reasonable.

NFATc1 is considered to be the key regulator of the terminal differentiation of osteoclasts. This factor is essential for the regulation of many osteoclast-specific genes, such as CTSK, TRAP, and CTR^41^,^42^. Here, we found that the expression of NFATc1 was significantly decreased at both the mRNA and protein levels following treatment with LY. LY treatment also inhibited the expression of TRAP, CTR, CTSK, c-Fos, DC-STAMP, V-ATPs-d2 and V-ATPs-a3 and therefore blocked osteoclast differentiation and bone resorption activity.

We further investigated the *in vivo* effect of LY in an OVX-induced osteoporosis model and a Ti particle-induced osteolysis model. The protective effect of LY was demonstrated by micro-CT analysis, H&E staining, and TRAP staining. RT-PCR for osteoclast-specific genes confirmed the role of LY in osteoclast formation *in vivo*. These results are the first to show that LY may be effective in the treatment of osteoporosis and wear particle-induced periprosthetic osteolysis. Nevertheless, our study suffers from certain weaknesses. For instance, MAPK overexpression may represent a better approach for the MAPK rescue experiment, as these results would further support our mechanistic results.

In conclusion, our results demonstrated the inhibitory effects of LY on osteoclastogenesis and osteoclast function both *in vitro* and *in vivo*. Our study also showed that LY mediated its effects through suppression of the P38, JNK, and ERK signalling pathways. Thus, our results indicate that LY may be developed as a potential treatment for osteoclast-related diseases.

## Methods

The animal experiments in this study were performed in accordance with the principles and procedures of the National Institutes of Health (NIH) Guide for the Care and Use of Laboratory Animals and the guidelines for animal treatment of Sir Run Run Shaw Hospital. All experimental protocols in this study were approved by the Ethics Committee of Sir Run Run Shaw Hospital.

### Cells, media, and reagents

The alpha modification of Eagle’s medium (α-MEM), penicillin/streptomycin, and foetal bovine serum (FBS) were purchased from Gibco-BRL (Gaithersburg, MD, USA). The cell counting kit (CCK-8) was obtained from Dojindo Molecular Technology (Kumamoto, Japan). Recombinant mouse M-CSF and mouse RANKL were obtained from R&D (Minneapolis, MN, USA), and LY was purchased from Sigma Aldrich (St Louis, MO, USA). Specific antibodies against ERK, JNK, P38, IκB-α, phospho-ERK (Thr202/Tyr204), phospho-JNK (Thr183/Tyr185), phospho-p38 (Thr180/Tyr182), MEK1/2, MKK6, MKK7, TAK1, phospho-MEK1/2, phospho-MKK6, phospho-MKK7, phospho-TAK1, NFATc1, and β-actin were obtained from Cell Signalling Technology (Cambridge, MA, USA). The TRAP staining kit, and all other reagents, were purchased from Sigma Aldrich unless otherwise stated.

### Mouse BMM preparation and osteoclast differentiation

Primary BMMs were isolated from the whole bone marrow of male, 6-week-old, C57BL/6 mice as described previously^27^,^43^. Cells were isolated from the femoral and tibial bone marrow and cultured in α-MEM supplemented with 10% FBS, 1% penicillin/streptomycin, and 30 ng/mL M-CSF in a 37 °C, 5% CO_2_ incubator until reaching 90% confluence. The BMMs were seeded into a 96-well plate at a density of 8 × 10^3^ cells/well, in triplicate, in the presence of 30 ng/mL M-CSF, 50 ng/mL RANKL, and different concentrations of LY (0, 0.1, 0.2 and 0.4 mM) with or without 2.5 pg/ml anisomycin (a P38 and JNK activator). The culture medium was replaced every 2 days until mature osteoclasts were formed. Then, the cells were washed twice with phosphate-buffered saline (PBS), fixed with 4% paraformaldehyde for 20 min, and stained for TRAP. TRAP-positive cells with more than five nuclei were counted under a microscope.

### Cell viability assay

The cytotoxic effects of LY on BMMs were determined using a CCK-8 assay. BMMs were plated in 96-well plates at a density of 2 × 10^4^ cells/well in triplicate in the presence of 30 ng/mL M-CSF for 24 h. Cells were then treated with different concentrations of LY (0, 0.1, 0.2, 0.4, 0.8, 1.6, 3.2 or 6.4 μM) for 48 or 96 h. Then, 10 μL of CCK-8 buffer was added to each well, and plates were incubated for an additional 2 h. The absorbance was measured at a wavelength of 450 nm (650 nm reference) on an ELX800 absorbance microplate reader (Bio-Tek Instr., Winooski, VT, USA).

### Resorption pit assay

BMMs were seeded at a density of 8 × 10^3^ cells/well onto bovine bone slices in a 96-well plate with three replicates. After 24 h, cells were treated with 50 ng/mL RANKL, 30 ng/mL M-CSF, and 0, 0.1, 0.2, or 0.4 μM LY until mature osteoclasts formed. Cells were then fixed with 2.5% glutaraldehyde. Resorption pits were visualized under a scanning electron microscope (FEI Instr., Hillsboro, OR, USA), and the bone resorption area was quantified using Image J software (National Institutes of Health, Bethesda, MD, USA).

### RNA extraction and quantitative RT-PCR assay

BMMs were seeded in 6-well plates at a density of 10 × 10^4^ cells/well and cultured in α-MEM supplemented with 30 ng/mL M-CSF, 50 ng/mL RANKL and 0.4 μM LY for 0, 1, 3 or 5 days. Total RNA was extracted using the RNeasy Mini kit (Qiagen, Valencia, CA, USA). Complementary DNA (cDNA) was synthesized using 1 μg of RNA from each sample, 2 μL of 5 × PrimeScript RT Master Mix (Takara Bio, Otsu, Japan), and 4 μL of RNase free dH_2_O in a total volume of 10 μL. RT-PCR was performed using an ABI Prism 7500 system (Applied Biosystems, Foster City, CA, USA) with SsoFast EvaGreen supermix (Bio-Rad, Hercules, CA, USA). The total volume (20 μL) of each PCR reaction consisted of 10 μL SsoFast EvaGreen supermix, 7 μL ddH_2_O, 2 μL cDNA, and 10 μM each of forward and reverse primers. The RT-PCR reaction was performed at 95 °C for 10 min (activation), followed by 40 cycles of 95 °C for 10 s, 60 °C for 20 s, and 72 °C for 20 s (amplification), and a final extension at 72 °C for 1 min, as previously described^44^. The quantity of each target was normalized to GAPDH. The mouse primer sequences were as follows:

GAPDH

forward 5′-ACCCAGAAGACTGTGGATGG-3′

reverse 5′-CACATTGGGGGTAGGAACAC-3′

CTSK

forward 5′-CTTCCAATACGTGCAGCAGA-3′

reverse 5′-TCTTCAGGGCTTTCTCGTTC-3′

CTR

forward 5′-TGCAGACAACTCTTGGTTGG-3′

and reverse 5′- TCGGTTTCTTCTCCTCTGGA-3′

TRAP

forward 5′-CTGGAGTGCACGATGCCAGCGACA-3′

reverse 5′-TCCGTGCTCGGCGATGGACCAGA-3′

c-Fos

forward 5′-CCAGTCAAGAGCATCAGCAA-3′

reverse 5′- AAGTAGTGCAGCCCGGAGTA-3′

NFATc1

forward 5′-CCGTTGCTTCCAGAAAATAACA-3′

reverse 5′-TGTGGGATGTGAACTCGGAA-30′

V-ATPase d2

forward 5′-AAGCCTTTGTTTGACGCTGT-3′

reverse 5′-TTCGATGCCTCTGTGAGATG-3′

V-ATPase a3

forward 5′-TGGCTACCGTTCCTATCCTG-3′

reverse 5′-CTTGTCCGTGTCCTCATCCT-3′

DC-STAMP

forward 5′-AAAACCCTTGGGCTGTTCTT-3′

reverse 5′-AATCATGGACGACTCCTTGG-3′

### Western blotting

To examine which signalling pathways were affected by LY, BMMs were seeded in 6-well plates at a density of 5 × 10^5^ cells/well. The cells were pre-treated with or without 0.4 μM LY for 2 h. Cells were then stimulated with 50 ng/mL RANKL for 0, 5, 10, 20, 30 or 60 min. To determine the effect of LY on NFATc1, BMMs were treated with 50 ng/mL RANKL, with or without 0.4 μM LY, for 0, 1, 3 or 5 days. Total protein was extracted from cultured cells using radioimmunoprecipitation assay (RIPA) lysis buffer (Sigma Aldrich, St Louis, MO, USA). Lysates were centrifuged at 12,000 × *g* for 15 min, and the supernatants were collected. Proteins were resolved on 10% SDS-PAGE gels and transferred by electroblotting to PVDF membranes (Bio-Rad, Hercules, CA, USA). The membranes were blocked in 5% nonfat dry milk in TBST (50 mM Tris (pH 7.6), 150 mM NaCl, 0.1% Tween 20) at room temperature for 1 h and then incubated with primary antibodies overnight at 4°C. Protein bands were developed using a horseradish peroxidase-conjugated goat anti-rabbit immunoglobulin G (Abcam, Cambridge, MA, USA), followed by detection using an electrochemical luminescence reagent (Millipore, Billerica, MA, USA). Protein bands were visualized using the LAS-4000 Science Imaging System (Fujifilm, Tokyo, Japan).

### OVX-induced bone-loss model

Twelve-week-old C57BL/6 female mice were generally anesthetized and subjected to either a sham operation or bilateral OVX. We randomly divided the mice into four groups: sham (sham operation and injection with PBS), vehicle (OVX and injection with PBS), low-dose LY (OVX and injection with 0.5 mg/kg LY), and high-dose LY (OVX and injection with 2.5 mg/kg LY). The mice were injected intraperitoneally with LY every other day for 6 weeks. All mice were sacrificed at the end of 6 weeks. Uteri were isolated and weighed to confirm the effects of OVX. Vertebral bodies of the lumbar spine (L3-L5) and left tibiae were fixed in 4% paraformaldehyde for micro-CT and histological studies. The right femurs and tibiae were frozen in liquid nitrogen immediately after dissection for subsequent total RNA extraction and quantitative RT-PCR.

### Ti particle-induced calvarial osteolysis model

A mouse calvarial osteolysis model was established using healthy male, 8-week-old, C57BL/6 mice. Mice were randomly assigned to four groups: sham (sham operation and injection with PBS), vehicle (Ti particle treatment and injection with PBS), low-dose LY (Ti particle treatment and injection with 0.5 mg/kg LY), and high-dose LY (Ti particle treatment and injection with 2.5 mg/kg LY). The mice were anesthetized, and the cranial periosteum was separated from the calvarium. Then, 30 mg of Ti particles were embedded under the periosteum at the middle suture of the calvaria. The mice were injected intraperitoneally with LY every other day for 2 weeks. At the end of the experiment, the mice were sacrificed, and calvaria were separated and fixed in 4% paraformaldehyde for micro-CT and histological analysis.

### Micro-CT scanning

The fixed calvaria, vertebral bodies, and tibiae were analysed using a high-resolution micro-CT scanner (Skyscan 1072; Skyscan, Aartselaar, Belgium). The scanning protocol was set at an isometric resolution at 9 mm and X-ray energy settings of 80 kV and 80 mA. BV/TV, BS/BV, Tb.Th., Tb.N., Tb.Sp and porosity were measured using the resident reconstruction program (Skyscan).

### Histological analysis

The fixed calvaria, vertebral bodies, and tibiae were decalcified in 10% EDTA for 3 weeks and then embedded in paraffin. Histological sections were prepared for TRAP and H&E staining. The specimens were then examined and photographed under a high-quality microscope. The number of TRAP-positive multinucleated osteoclasts per field and OcS/BS were examined in each sample using Image-Pro Plus software (Media Cybernetics, Bethesda, MD, USA).

### Statistical analysis

The data were expressed as means ± SEM (standard error of the mean). Experiments were conducted separately at least three times. The results were analysed using SPSS for Windows, version 16.0 (SPSS, Chicago, IL, USA). The student’s t-test was used to make comparisons between two groups. *P* < 0.05 indicated a significant difference between groups.

## Additional Information

**How to cite this article**: Chen, S. *et al.* Lycorine suppresses RANKL-induced osteoclastogenesis *in vitro* and prevents ovariectomy-induced osteoporosis and titanium particle-induced osteolysis *in vivo*. *Sci. Rep.* **5**, 12853; doi: 10.1038/srep12853 (2015).

## Supplementary Material

supplemental figure S1
