# Supplementary material for: Lycorine suppresses RANKL-induced osteoclastogenesis in vitro and prevents ovariectomy-induced osteoporosis and titanium particle-induced osteolysis in vivo
Source: Sci Rep. 2015 Aug 4;5:12853. doi: 10.1038/srep12853 (PMC4523876; doi:10.1038/srep12853)
Supplement: supplemental figure S1 [file srep12853-s1.pdf]

Supplemental Figure S1

TITLE: Lycorine suppresses RANKL-induced osteoclastogenesis in vitro and prevents ovariectomy-induced osteoporosis and titanium particle-induced osteolysis in vivo

AUTHORS: Shuai Chen, Gu Jin, Kang-Mao Huang, Jian-Jun Ma, Qiang Wang, Yan Ma, Xiao-Zhen Shen, Zhi-Jie Zhou, Zhi-Jun Hu, Ji-Ying Wang, An Qin, Shun-Wu Fan

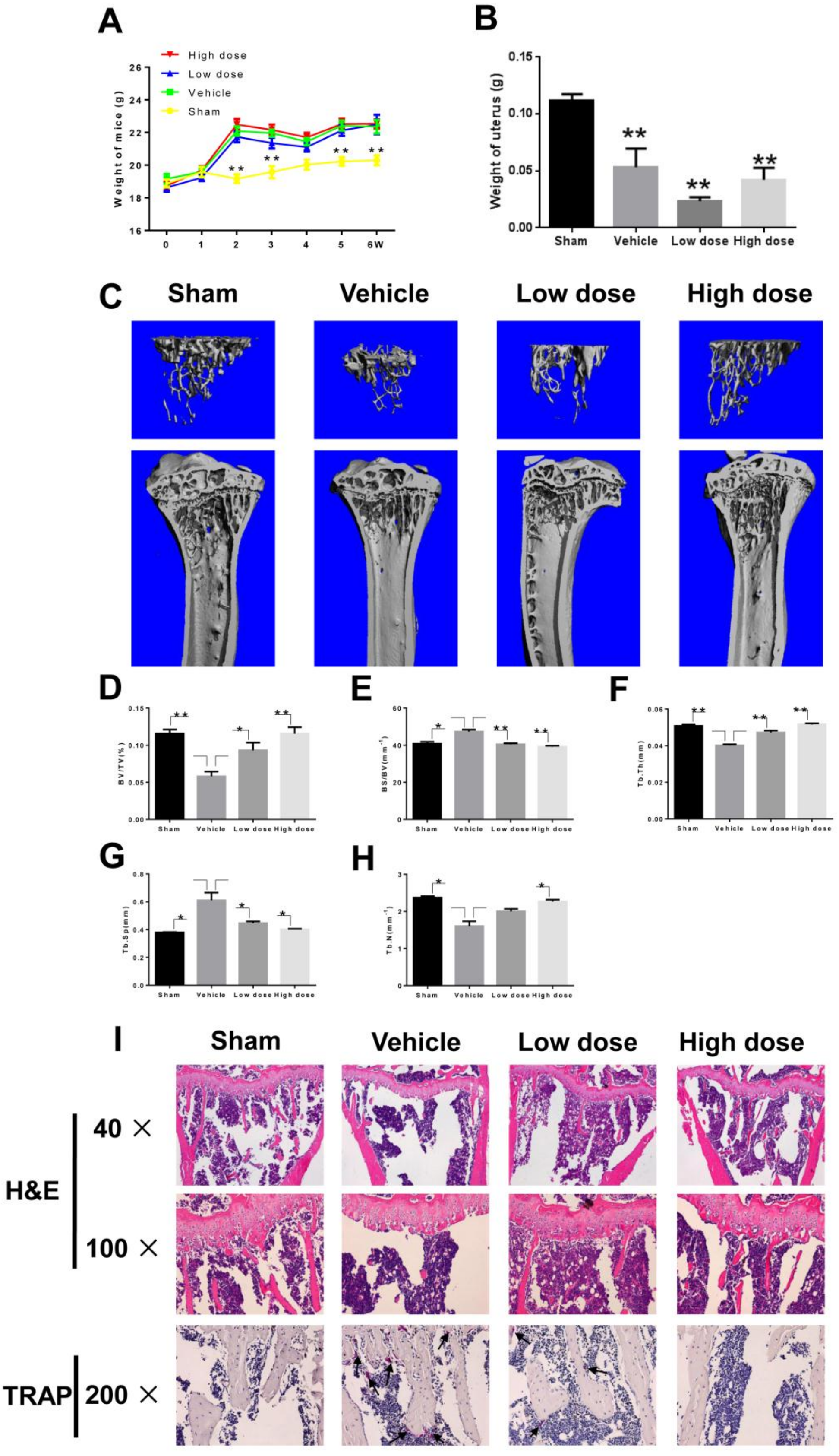
A
High dose
Low dose
Vehicle
Sham
Weight of mice (g)
**
6W
B
Weight of uterus (g)
Sham
Vehicle
Low dose
High dose
C
Sham
Vehicle
Low dose
High dose
D
BV/TV(%)
E
BS/BV(mm-1)
F
Tb.Th(mm)
G
Tb.Sp(mm)
H
Tb.N(mm-1)
I
Sham
Vehicle
Low dose
High dose
H&E
40 ×
100 ×
TRAP
200 ×

**Fig. S1.** LY effectively prevents OVX-induced bone loss *in vivo*. (A): The body weight of mice 0-6 weeks after OVX in Sham, Vehicle, Low dose and High dose groups (**$P$ < 0.01). (B): The weight of the mouse uteri at the time of sacrifice in all four groups (**$P$ < 0.01). (C): The left tibiae of all mice were scanned with a high-resolution micro-CT. (D)-(H): The calculation of the microstructural indices was performed for the micro-CT data as described in the Methods section. Microstructural indices include bone volume/tissue volume (BV/TV), bone surface/bone volume (BS/BV), trabecular separation (Tb.Sp.), trabecular thickness (Tb.Th.), and trabecular number (Tb.N.) (*$P$ < 0.05; **$P$ < 0.01). (I): the tibiae were fixed in 4% paraformaldehyde, then decalcified, embedded and sectioned as described in the Methods section. Sections of tibiae were stained with H&E (40× and 100×) and TRAP (100× and 200×).
